# The Relationship Between Family Accommodation and Anxiety Sensitivity in Obsessive-Compulsive Disorder

**DOI:** 10.7759/cureus.43793

**Published:** 2023-08-20

**Authors:** Rıza Gökçer Tulacı, Meltem İzci Kasal

**Affiliations:** 1 Department of Psychiatry, Balıkesir University, Balikesir, TUR; 2 Psychiatry, Balıkesir Atatürk City Training and Research Hospital, Balikesir, TUR

**Keywords:** ocd, compulsion, obsession, family accommodation, anxiety sensitivity, : obsessive compulsive disorder

## Abstract

Objective

Anxiety sensitivity (AS) is an issue that has gained importance in obsessive-compulsive disorder (OCD) in recent years. Family accommodation (FA) is the participation of family members in compulsions and rituals. The objectives of this study were to (1) investigate the relationship between FA and AS in OCD and (2) determine the frequency and types of FA in a Turkish OCD sample.

Methods

This cross-sectional study was conducted with 81 OCD patients. The Yale-Brown Obsession Compulsion Scale (YBOCS), Family Accommodation Scale-Patient Form (FAS-PF), Anxiety Sensitivity Index-3 (ASI-3), Beck Depression Inventory (BDI), and Beck Anxiety Inventory (BAI) were administered to the patients.

Results

Family accommodation was extremely prevalent among family members of OCD patients. There were significant relationships between FA and ASI-3 total, ASI-3 cognitive subscale, ASI-3 psychical subscale, YBOCS, and BAI scores. In addition, ASI-3 total, ASI-3 cognitive subscale, and ASI-3 psychical subscale were significant predictors for family accommodation above and beyond the OCD severity.

Conclusions

The present study identified a significant association between anxiety sensitivity and family accommodation in OCD. Anxiety sensitivity is a relievable psychological trait. Alleviating anxiety sensitivity can decrease accommodating behaviors and may be beneficial in OCD treatment. Anxiety sensitivity may be a novel potential treatment target for OCD.

## Introduction

Obsessive-compulsive disorder (OCD) is a chronic mental illness characterized by obsessions and compulsions that are common all over the world. Obsessive-compulsive disorder is time-consuming and causes clinically significant distress and impairment in social, occupational, or other important areas of functioning [1]. There are various types of obsessions and compulsions that are spread over many aspects of daily life. Therefore, OCD affects the functioning of family members as well as the patient [2].

Obsessions cause intense anxiety and distress, and the patients perform compulsions or avoidance behaviors to reduce this distress. Relatives of the patient who witness the intense distress and anxiety felt by the patient may facilitate, allow, or sometimes participate in compulsions, rituals, or avoidance behaviors in order to alleviate the negative experiences and distress of the patient related to the disorder. This phenomenon seen in OCD is called family accommodation (FA). Family accommodation can occur in several ways. Family members may actively participate in compulsions, for example, by cleaning unpolluted surfaces together with the patient. They can prevent the triggering stimuli that elicit obsessions by taking the clothes to a specific place requested by the patient without touching anything when they come from outside. They do not interfere with the compulsions by ignoring the patient washing his or her hands for a long time.

Family accommodation is highly prevalent among the relatives of patients with OCD, with rates ranging from 62% to 100% [3]. Although FA may reduce the patient's stress for a very short time, it ultimately has a significant negative impact on the course of OCD. Family accommodation prevents the patient from confronting their obsessive thoughts and the anxiety triggered by these obsessions. Therefore, FA hinders the effectiveness of exposure and response prevention. It has been reported that there is a relationship between higher family accommodation levels and worse treatment outcomes in OCD [4]. Previous studies showed that severe FA was associated with severe OCD symptoms and functional impairment [2,5]. Thus, investigating potential aspects that may be related to FA is important for a better understanding of this treatment-resistant disease and for identifying novel treatment targets in OCD. The presentation of OCD symptoms and family responses to these symptoms may also vary across cultures. There is only one paper investigating family accommodation in OCD in Turkish culture. Kuru et al. reported that all the family members participating in their study exhibited at least one accommodating behavior in a one-week period, and contamination-type obsessions were associated with accommodating behavior in this paper [6]. Our study may also contribute to the literature in terms of adding new data on the manifestation of FA in different cultures.

Anxiety sensitivity (AS) is conceptualized as a feature that reflects an individual's propensity to fear the consequences of anxiety or anxious arousal. Because the person believes that anxiety or anxious arousal can lead to physical, psychological, or socially harmful results [7], a person with increased AS may misinterpret chest tightness as a sign of a heart attack, racing thoughts as a manifestation that one is "going crazy", and experiencing anxiety symptoms as something to be ashamed of [8].

Anxiety sensitivity has historically been examined within the context of anxiety disorders. There are persuasive results indicating that AS plays a crucial role in the onset and persistence of several anxiety disorders, including panic disorder, social anxiety disorder, and post-traumatic stress disorder. There has been a growing body of literature on the importance of AS in OCD in recent years after studies related to anxiety disorders. Several studies have demonstrated that OCD patients have greater levels of AS compared to healthy individuals and that greater AS is significantly associated with severe OCD symptoms and poor treatment outcomes [8,9]. In addition, studies have revealed associations between subdomains of AS (physical concern, cognitive concern, and social concern) and different OCD symptom dimensions (for example, AS physical concern was a significant predictor for contamination symptoms, AS social and cognitive concerns were significant predictors of unacceptable thoughts, symmetry dimension, etc.) [9]. However, studies on AS and FA in OCD are scarce. In addition, the very limited data available on this topic are inconsistent. Wu et al. reported a significant relationship between AS and FA, and the cognitive concern subfactor predicted FA severity, whereas Cosentino et al. found that AS was not a predictor of FA [2,10]. So, the relationship between AS and FA anxiety sensitivity is an important subject in the OCD field that is understudied.

The aim of this study was to (1) examine the relationship between FA and AS in OCD and (2) determine the frequency and types of FA in a Turkish OCD sample. We hypothesize that there would be a significant relationship between family accommodation and anxiety sensitivity in obsessive-compulsive disorder. We also hypothesize that family accommodation would be very prevalent among the family members of OCD patients.

## Materials and methods

Participants and procedures

The present study was conducted with patients diagnosed with OCD admitted to the psychiatry outpatient clinic of Balıkesir Atatürk City Hospital, a tertiary care hospital in Balıkesir, Turkey. Ninety-four patients aged 18-65 who volunteered to participate in the study and who had been living with a relative older than 18 years old for at least six months were enrolled in the study. The exclusion criteria were as follows: schizophrenia/psychotic disorders; bipolar disorder; active alcohol and substance use disorder; mental retardation; a severe neurological disorder that may interfere with the scale results (stroke, head trauma, etc.); and the diagnosis of any mental illness in the family member evaluated for FA. Thirteen patients were excluded from the study (three patients had bipolar disorder, two patients had a psychotic disorder, one patient had an alcohol-use disorder, and seven patients had a family member with a mental illness). Finally, the study was carried out with 81 patients.

A psychiatrist with expertise in OCD administered the clinician version of the Structured Clinical Interview for The Diagnostic and Statistical Manual of Mental Disorders, Fifth Edition (DSM-5) Disorders (SCID-5-CV) [11] to patients and their family members to confirm the diagnosis of OCD and to identify psychiatric disorders. The Yale-Brown Obsession Compulsion Scale (YBOCS), Family Accommodation Scale-Patient Form (FAS-PF), Anxiety Sensitivity Index-3 (ASI-3), Beck Depression Inventory (BDI), and Beck Anxiety Inventory (BAI) were administered to the patients.

Informed consent was obtained from all participants. Ethics committee approval was obtained from the Ethics Committee of Balıkesir Atatürk City Hospital (date and number: 2023/4/35).

Measures

The Yale-Brown Obsession Compulsion Scale (YBOCS)

The Yale-Brown Obsession Compulsion Scale is a semi-structured instrument measuring the severity of OCD. Five items measure the severity of obsessions, and five items measure the severity of compulsions. Each item is scored between 0 and four. The sum of 10 items indicates the severity of the disorder. Higher scores indicate severe OCD symptoms [12]. Turkish adaptation, validity, and reliability were performed by Tek et al. [13].

Family Accommodation Scale for Obsessive-Compulsive Disorder, Patient Version (FAS-PV)

It is a self-report scale that assesses the nature and frequency of family accommodation behaviors in OCD. It consists of two parts. The first part is a symptom checklist that provides information about OCD symptoms. Part two consists of 19 items that assess the type and frequency of accommodating behaviors of family members in the last week. Items are rated by the patient in terms of frequency on a scale of 0-four (0: none, four: every day). The total score is obtained by summing the 19 items in Part Two. High scores indicate severe accommodating behaviors [14]. The Turkish adaptation study of the scale was performed by Çöldür et al. [15].

Anxiety Sensitivity Index-3 (ASI-3)

It is a self-report questionnaire that measures anxiety sensitivity multi-dimensionally. The ASI-3 consists of 18 items with physical, cognitive, and social subdimensions, of which each subdimension has six items. Cognitive concerns are associated with the belief that concentration difficulties or overspeeding of thoughts lead to insanity. Social concerns are associated with the belief that manifestations of anxiety reactions in public (e.g., intense tremor) lead to social rejection or derision. Physical concerns are associated with the misinterpretation of anxiety-related bodily sensations as causing catastrophic physical illnesses. Each item is a 5-point Likert-type measure scored between 0 and four. The score that can be obtained from the scale is between 0-72. High scores on the scale indicate increased anxiety sensitivity [16]. A Turkish validity and reliability study was conducted by Mantar et al. [17].

Beck Depression Inventory (BDI)

The BDI is a 21-item self-report scale that measures the severity of depressive symptoms. Each item is scored between 0 and three, and the total score ranges from 0 to 63. A higher total score indicates more severe depression symptoms [18].

Beck Anxiety Inventory (BAI)

This is a 21-item self-report scale that measures the level of anxiety symptoms. Each item is scored from 0 to three, and the total score ranges from 0 to 63. Higher total scores indicate more severe anxiety symptoms [19].

Statistical analysis

Data were analyzed using IBM SPSS Statistics software for Windows, Version 26.0 (Armonk, NY: IBM Corp.). Descriptive statistics were expressed as the mean ± standard deviation, number, and percentage. The normal distribution was tested using the Z score, histograms, and normal probability plots (q-q plots). A chi-square test was used to analyze categorical variables. Pearson’s correlation analysis was used to evaluate the correlations between family accommodation and various clinical variables. A multivariate hierarchical regression analysis was performed to determine whether AS was a predictor of FA. Statistical significance was set at p < 0.05.

## Results

The present study was conducted with 81 patients diagnosed with OCD. The mean age was 34,5 ± 11,7 years, with 53.1% (n = 43) of the participants being female; 55.6% (n = 45) of the patients were married; and the mean period of education was 10.5 ± 3.5 years. The demographic and clinical characteristics of the patients are presented in Table 1.

**Table 1:** Demographic and clinical characteristics of patients n: number; Sd: standard deviation; FAS: Family Accommodation Scale; Y-BOCS: Yale-Brown Obsession Compulsion Scale; ASI-3: Anxiety Sensitivity Index-3; BDI: Beck Depression Inventory; BAI: Beck Anxiety Inventory

n=81	Mean ± Sd / n (%)
Age (years)	34.5 ±11.7
Gender (women)	43 (53.1)
Marital status (married)	45 (55.6)
Educational level (years)	10.5 ± 3.5
Age at OCD onset	18,3 ± 7,6
Type of obsessions	
Contamination	45 (55,6)
Aggressive	9 (11,1)
Sexual	8 (9,9)
Religious	15 (18,5)
Symmetry	18 (22,2)
Somatic	9 (11,1)
Hoarding	4 (4,9)
Miscellaneous	28 (43,6)
Type of compulsions	
Cleaning/washing	43 (53,1)
Repeating	11 (13,6)
Arranging/ordering	15 (18,5)
Counting	19 (23,5)
Hoarding	6 (7,4)
Miscellaneous	23 (28,4)
Y-BOCS-Total	23,8 ± 7,8
Y-BOCS: Obsessions	11,9 ± 3,7
Y-BOCS: Compulsions	11,9 ± 4,3
FAS-PV	20,4 ± 15,2
Family members	
Spouses	40 (49,4)
Parents	25 (30,9)
Offspring	12 (14,8)
Others	4 (4,9)
ASI-3 Total	23,7 ± 9,5
ASI-3-Physical	7,4 ± 4,5
ASI-3-Cognitive	9,2 ± 4,9
ASI-3-Social	7,0 ± 5,0
BDI	17,4 ± 12,5
BAI	19,6 ± 11,1

There were 77 (95.1%) family members who engaged in at least one accommodating behavior in the previous week. There were 31 (39.5%) family members who engaged in at least one accommodating behavior every day. The most common family-accommodating behaviors in our sample were providing reassurance, waiting for compulsions, tolerating OCD behaviors, and facilitating compulsions (Table 2).

**Table 2:** Frequency and distribution of accommodation behavior among family members a: Minimum score of one on any accommodating behavior (an individual who exhibited at least one accommodation behavior in the previous week), b: At least one of the questions with a score of four (an individual who performed at least one adaptive behavior on a daily basis in the previous week).

n:81	Prevalence^a^ n (%)	Daily /Extreme^b^ n (%)
1. Providing reassurance (obsession)	66 (81.5)	18 (22.2)
2. Providing reassurance (compulsions)	38 (46.9)	7 (8.6)
3. Waiting for compulsions	52 (64.2)	14 (17.3)
4. Participating in compulsions	36 (44.4)	8 (9.9)
5. Facilitating compulsions	54 (65.7)	9 (11.1)
6. Providing items for performing compulsions	33 (40.7)	1 (1.2)
7. Facilitating avoidance	46 (56.8)	5 (6.2)
8. Helping decisions	33 (40.7)	2 (2.5)
9. Helping with personal tasks	33 (40.7)	5 (6.2)
10. Helping prepare food	34 (42.0)	3 (3.7)
11. Taking on family or household responsibilities	44 (54.3)	3 (3.7)
12. Avoiding talking about things that might trigger obsessions or compulsions	41 (50.6)	2 (2.5)
13. Stopping himself/herself from doing some things	44 (54.3)	8 (9.9)
14. Excusing/lying	16 (19.8)	0
15. Tolerating	51 (63.0)	7 (8.6)
16. Putting up with	39 (48.1)	11 (13.6)
17. Cutting back on leisure activities	28 (34.6)	2 (2.5)
18. Modifying personal routine	26 (32.1)	3 (3.7)
19. Putting off some of his/her family responsibilities	35 (43.2)	9 (11.1)
Total	77 (95.1)	31 (39.5)

To examine the relationship between FA and variables of interest, Pearson’s correlation analysis was conducted between the FAS-PV score and variables including Y-BOCS, ASI-3 Total, ASI subscales, BDI, and BAI. The FAS-PV score was significantly correlated with ASI-3 total, ASI-3-physical subscale, ASI-3-cognitive subscale, Y-BOCS total, Y-BOCS obsessions, Y-BOCS compulsions, and BAI scores. The FAS-PV did not exhibit a significant correlation with the ASI-3-social subscale. Higher scores on the ASI-3 (total, cognitive, and physical subscale scores) were related to higher scores on family accommodation (Table 3).

**Table 3:** Correlations among study measures r: Pearson correlation coefficient; * = p<0.05, **= p < 0.01; FAS-PV: Family Accommodation Scale; Y-BOCS: Yale-Brown Obsession Compulsion Scale; ASI-3-T: Anxiety Sensitivity Index-3 total; ASI-3-C: ASI-3-cognitive subscale; ASI-3-P: ASI-3-physical subscale; ASI-3-S: ASI-3-social subscale; YBOKÖ-O: Y-BOCS obsessions, YBOKÖ-C: Y-BOCS compulsions; YBOKÖ-T: Y-BOCS total; BDI: Beck Depression Inventory; BAI: Beck Anxiety Inventory

n=81	1	2	3	4	5	6	7	8	9	10
	r	r	r	r	r	r	r	r	r	r
1. FAS-PV	1									
2. ASI-3-T	0.622^**^	1								
3. ASI-3-C	0.650^**^	0.752^**^	1							
4. ASI-3-P	0.668^**^	0.763^**^	0.588^**^	1						
5. ASI-3-S	-0.062	0.467^**^	-0.091	-0.031	1					
6.YBOKÖ-O	0.685^**^	0.335^**^	0.374^**^	0.347^**^	-0.046	1				
7. YBOKÖ-C	0.693^**^	0.290^**^	0.408^**^	0.321^**^	-0.142	0.885^**^	1			
8.YBOKÖ-T	0.704^**^	0.323^**^	0.407^**^	0.346^**^	-0.101	0.957^**^	0.968^**^	1		
9. BAI	0.568^**^	0.402^**^	0.434^**^	0.306^**^	0.059	0.555^**^	0.456^**^	0.521^**^	1	
10. BDI	0.93	0.171	0.135	0.100	0.101	0.294^**^	0.249^*^	0.280^*^	0.190	1

We next performed a series of multivariate hierarchical regression analyses with the Enter method to assess the association between family accommodation and anxiety sensitivity after controlling for OCD severity. Two separate regression models were executed for the ASI-3 total and ASI-3 subscales. The FAS was included in the analysis as a dependent variable, and the YBOCS-T was included as a covariate in the first step. In the second step, ASI-3 total, or ASI-3 subscales (cognitive subscale, social subscale, physical subscale), and BAI scores, which were found significant in bivariate analyses, were added to the model as independent variables. Preliminary analyses of regression models indicated no threats to normality, multicollinearity, or homoscedasticity.

Model 1: In the first step, the YBOCS explained a significant proportion of variance in FAS scores (coefficient of determination (R^2^): 0.49, p < 0.001). In the second step, adding the ASI-3 total and BAI significantly increased the variance accounted for (R^2^ change: 0.18, p < 0.001). The final model accounted for 68% of the variance and was significant (R^2^:0.68, p < 0.001). In the final model, ASI-3 total and YBOCS total emerged as significant individual predictors for family accommodation. In addition, ASI-3 total was a significant predictor for family accommodation above and beyond OCD symptom severity (Table 4).

**Table 4:** Regression analysis for predicting family accommodation in OCD patients R^2^: coefficient of determination; Std: standard; Sig: statistical significance; Family Accommodation Scale (FAS): dependent variable; Model 1; ANOVA: p<0.001; Model 2; ANOVA: p<0.001; Y-BOCS-T: Yale-Brown Obsession Compulsion Scale - Total; ASI-3-T: Anxiety Sensitivity Index-3 total; ASI-3-C: ASI-3-cognitive subscale; ASI-3-P: ASI-3-physical subscale; ASI-3-S: ASI-3-social subscale; BAI: Beck anxiety inventory; OCD: obsessive compulsive disorder

			B	Std. Error	Beta	Sig	R^2^
Model 1	Step 1	Constant	-12.201	3.902		0.002	0.49
		YBOCS-T	1.370	0.156	0.704	<0.001	
	Step 2	Constant	-21.814	3.498		<0.001	0.68
		YBOCS-T	0.967	0.148	0.497	<0.001	
		ASI-3 Total	0.639	0.112	0.402	<0.001	
		BAI	0.203	0.108	0.148	0.063	
Model 2	Step 1	Constant	-12.201	3.902		0.002	0.49
		YBOCS-T	1.370	0.156	0.704	<0.001	
	Step 2	Constant	-17.780	3.272		<0.001	0.75
		YBOCS-T	0.813	0.137	0.417	<0.001	
		ASI-Cognitive	0.619	0.233	0.203	0.010	
		ASI-Psychical	1.200	0.242	0.357	<0.001	
		ASI-Social	0.003	0.176	0.001	0.987	
		BAI	0.211	0.098	0.154	0.054	

Model 2: In the first step, the YBOCS explained a significant proportion of the variance in FAS scores (R^2^:49, p < 0.001). In the second step, adding the ASI-3 cognitive, physical, and social subscale scores and BAI significantly increased the variance accounted for (R^2^ change: 0.25, p < 0.001). The final model accounted for 75% of the variance and was statistically significant (R^2^: 0.75, p < 0.001). In the final model, ASI-3 physical, ASI-3 cognitive subscales, and YBOKS total emerged as significant individual predictors of family accommodation. The ASI-3 physical and ASI-3 cognitive subscales specifically were a predictor of family accommodation after controlling for OCD severity (Table 4).

## Discussion

The present study examines the relationship between AS and FA in OCD. It also investigated the frequency and nature of FA in the Turkish population with OCD. We found that ASI-3 total, ASI-3 cognitive subscale, ASI-3 psychical subscale, and OCD severity scores were associated with FA severity and that these variables were predictors of FA. Moreover, ASI-3 total, ASI-3 cognitive subscale, and ASI-3 psychical subscale were significant predictors for family accommodation above and beyond the OCD symptom severity. When the frequency of FA behaviors was assessed, 95% of family members exhibited at least one accommodating behavior in the previous week, and 39.5% of them exhibited any type of accommodating behavior on a daily basis or extreme in a one-week period.

Our study found that accommodating behaviors were extremely prevalent among family members of OCD patients. The most frequently accommodating behaviors were providing reassurance and facilitating compulsions. Several studies have reported that FA is very common in the relatives of OCD patients, with a prevalence of up to 97%, and that providing reassurance, waiting for compulsions, and tolerating rituals are the most common accommodating behaviors [20,21]. Specifically, Kuru et al. reported that all family members exhibited at least one accommodating behavior in a one-week period, and the most common adaptation behavior was providing reassurance in a population of Turkish patients with OCD [6]. Our results are consistent with previous studies and extend the data in different cultures about family accommodation in OCD. Clinicians should be aware of this common phenomenon, which has negative consequences, including treatment refractoriness and negative family functioning [22]. Interventions to reduce these behaviors may improve treatment outcomes for OCD.

Higher levels of family accommodation were associated with greater OCD severity in our study. Many previous studies on the relationship between FA and OCD have reported similar results to our findings [21,22]. However, it remains controversial whether severe OCD symptoms increase FA or whether severe accommodating behaviors exacerbate OCD symptoms. Relatives of patients who observe intense and prolonged rituals resulting from severe OCD symptoms may engage in more accommodating behaviors to relieve the patient's emotional stress and anxiety. On the other hand, intense accommodating behaviors, which have a disruptive effect on OCD treatment [23], may also exacerbate OCD symptoms by interfering with OCD treatment. Further studies that explore the underlying mechanisms of this relationship between OCD severity and FA will provide more enlightening data.

In our study, there was a significant positive correlation between FAS-PV and ASI-3 total and two subdomains of ASI-3, including AS3-physical concern and AS3-cognitive concern. Furthermore, AS3-total, AS3-physical subscale, and AS3-cognitive subscale remained significant predictors of family accommodation even after controlling for OCD severity. The association between AS and FA is not affected by psychopathology in relatives, as we administered the SCID-5-CV to family members and excluded those diagnosed with any mental illness from the study. This refined result is one of the strengths of our study.

Wu et al. found that there were positive correlations between family accommodation and anxiety sensitivity in patients with OCD. In addition, they reported that ASI-total and ASI cognitive subscales were predictors of FA [2]. Although our study is largely consistent with their results, there is a difference between the two studies in terms of the ASI-3 physical subscale. They did not find the ASI physical subscale to be a predictor of FA. This difference may be due to using different scales. They used the ASI scale, which has a four-factor structure, to measure anxiety sensitivity, whereas we used the ASI-3 scale with three factors. Patients with severe AS are more vulnerable to stress, anxiety, and anxiety-related sensations caused by OCD [2,8]. Further, they may experience these symptoms more seriously than those without severe AS. Storch et al. suggested that patients with high AS have significant difficulty resisting obsessions and controlling compulsions [24]. Severe AS also has a negative impact on both the treatment and prognosis of OCD [2,8]. Cognitive behavioral therapy with exposure and response prevention (ERP) is an effective treatment for OCD [25]. Exposure involves repeated, systematic confrontation with situations and stimuli that trigger obsessive anxiety. It has been suggested that elevated AS enhances the difficulty of ERP and that fear of anxious arousal predicts a poor treatment outcome for OCD [7]. Therefore, severe AS can exacerbate compulsions, rituals, and obsessions. Family members who witness the severe anxiety, stress, sensitivity to anxiogenic stimuli, and prolonged rituals experienced by patients may perform more accommodating behaviors. Furthermore, higher family accommodation levels lead to worse treatment results for OCD (4). It has been shown that anxiety sensitivity exacerbates FA and thus causes functional impairment in OCD in a study using mediator analysis [2]. Anxiety sensitivity could be a potential target for OCD. The brief AS intervention was effective in reducing overall AS, and these reductions were maintained over two years [26]. Therefore, reducing AS may have the additional benefit of improving OCD symptoms both directly and indirectly by also lowering FA.

It was found that the ASI-3 physical subscale and ASI-3 cognitive subscale were associated with the contamination, responsibility for harm, and symmetry dimensions of OC symptoms [9,27]. In our study, these two subdomains of AS were predictors of FA severity. The OCD symptoms related to these dimensions (excessive cleaning, quickly running out of cleaning materials, problems with items used in common with family members such as door handles, TV remotes, etc., repetitive door and stove checks, being unsure and asking repetitive questions, organizing and arranging household items, etc.) significantly affect the daily lives of family members. These symptoms, which affect both the patient and their relatives, may exacerbate family accommodations.

The present study's findings should be considered in relation to its limitations. First, the cross-sectional design prevents establishing a causal relationship exactly. Second, the study was performed in a psychiatry outpatient clinic of a tertiary care hospital, which prevents the generalization of the results to the entire OCD population. Third, we used a method similar to most studies on FA for selecting family members. The individual who participated the most in accommodating behaviors and spent the most amount of time with the patient was evaluated for FA, while all other family members were excluded. This could have affected the results of the study. Fourth, in addition to semi-structured measurement tools, self-report measurement tools were also used in the study. The use of self-report scales was another limitation of the study. Finally, the lack of a control group and the non-standardization of the treatments administered to the patients were also limitations of the study. Future prospective studies with larger samples examining the therapeutic effect of interventions to reduce anxiety sensitization on FA and OCD may provide new insights for OCD treatment.

## Conclusions

In conclusion, despite these limitations, the current study identified a significant relationship between anxiety sensitivity and family accommodation in OCD. Severe anxiety sensitivity was correlated with both severe family accommodation and severe OCD symptoms. Moreover, AS total and two subdomains (AS cognitive concern and AS physical concern) were independent predictors of FA even after controlling for OCD severity. Anxiety sensitivity is a highly malleable construct and could be a novel potential target for OCD treatment. Alleviating AS can facilitate ERP and decrease accommodating behaviors. Reducing AS may be beneficial for the treatment of OCD symptoms, both directly and indirectly, by attenuating FA.
